# Financial costs of pediatric cancer management in Africa: systematic review

**DOI:** 10.3389/fpubh.2023.1175560

**Published:** 2023-09-22

**Authors:** Criss Koba Mjumbe, Desiré Mashinda Kulimba, Oscar Luboya Numbi, Murielle Nkumuyaya, Diane Muatama Balimo, Chadrack Kabeya Diyoka, Benjamin Kabyla Ilunga

**Affiliations:** ^1^Department of Public Health, Faculty of Medicine, University of Lubumbashi, Lubumbashi, Democratic Republic of Congo; ^2^Department of Public Health, School of Public Health, University of Lubumbashi, Lubumbashi, Democratic Republic of Congo; ^3^Department of Public Health, School of Public Health, University of Kinshasa, Kinshasa, Democratic Republic of Congo; ^4^Department of Paediatrics, Faculty of Medicine, University of Lubumbashi, Lubumbashi, Democratic Republic of Congo; ^5^Rosalind Franklin Laboratory, Coventry, United Kingdom

**Keywords:** financial costs, Africa, systematic review, cancer, pediatric

## Abstract

The high costs of cancer treatment and the lack of investment in health care are significant barriers to public health on the African continent. The objective of this study was to investigate the financial cost of children cancer treating in sub-Saharan Africa. We systematically searched PubMed, Cochrane, and Google Scholar to identify relevant studies between March 2000 and December 2022. We selected articles that specifically addressed the US dollar financial costs of childhood cancer in African countries. Medians and interquartile ranges (IQR) were calculated. We also calculated the economic burden of childhood cancer at the individual level, by dividing the direct costs of cancer per patient by the GDP *per capita*, PPP of the country studied. The quality of economic studies was assessed using the CHEERS (2022) 28-point checklist. A total of 17 studies met our eligibility criteria. The median (IQR) of total childhood cancer costs by region was $909.5 ($455.3–$1,765) and ranged from $88803.10 for neuroblastoma to $163.80 for lymphoma. No significant differences (*p* < 0.05) were observed for comparisons of the direct cost of childhood cancer between the geopolitical zone of sub-Saharan Africa. Differences in the direct costs of childhood cancer were significant for different cancer types (*p* < 0.05). In the majority of 17 out of 54 countries on Africa the continent, the economic burden of childhood cancer exceeds 80% of GDP *per capita*, PPP, up to 345.38% of Nigeria’s GDP for Rhabdomyosarcoma. The cost of treating childhood cancers is high in Africa is catastrophic, if not downright prohibitive for households in Sub-Saharan Africa. We believe that the data from our study will be able to help make different objective advocacy allowing it to be provided with funds based of the evidence that can strengthen this program in order to install cancerology structures in the countries and by following the system plan. Cost reduction in the treatment of childhood cancer in particular and in general all types of cancer.

**Systematic review registration:**

Approval of the study was given by the ethics committee of the Faculty of Medicine of the University of Lubumbashi (UNILU/CEM/135/2018) and (UNILU/CEM/096/2019).

## 1. Introduction

Previously, cancer was considered as the health problem related to high-income countries. Nowadays, cancer no longer spares Africa, where the numbers of new cases and deaths are sky rocketing (a nearly 100% increase is expected by 2030) (1). The high costs of cancer treatment and lack of investment in health care are significant obstacles to public health on the African continent. African countries pledged through the Abuja declaration to allocate 15% of their gross domestic product to the healthcare sector. However, that objective has not been reached (2). In most African countries, patients bear a high percentage of healthcare expenses (3, 4). Public health spending on the continent has mainly targeted infectious and parasitic diseases (AIDS, malaria, tuberculosis, etc.) and not cancer. And public aid from developed countries has similarly targeted epidemics, such as the Ebola virus and other crises, leaving the fight against cancer relegated to the background (5, 6). According to the Global Task Force on Expanded Access to Cancer Care and Control, only 5% of the world’s cancer resources are spent in developing countries, and individual countries must draw up their own multi-year cancer plans adapted to their own socio-economic situations (7). To our knowledge, no researcher has specifically estimated the costs of treating pediatric cancers in Africa.

Researchers, clinicians and families know that cancer is a costly disease. Health professionals and policy makers in the health system at both national and regional level need data on the costs of cancer in general and those relating to childhood and adolescent cancer treatment in order to determine a distribution of health resources that meets to the needs of families, and better alignment in the adaptation of available resources. According to the Global Task Force on Expanded Access to Cancer Care and Control, only 5% of global cancer resources are spent in developing countries, and each country must develop its own multi-year cancer control plans adapted to their own socio-economic situation (7). Thus, our objective with this study was to help with estimating the financial costs of pediatric cancers in Sub-Saharan Africa based on the operational definitions of Heinrich (8–10).

## 2. Methods

This is a systematic review, the Preferred Reporting Items for Systematic Reviews and Meta-Analysis (PRISMA) guidelines (11), were adopted and the PRISMA checklist followed. The study was based on the operational definitions of Heinrich (8–10), who defines direct costs (DC) as current financial burdens attributable to disease acts, including hospitalization costs, medical care and laboratory costs, while indirect costs (CI) represent costs in terms of time and other resources (time paid and not by work, time lost, damage caused, interruption of production, social charges, loss of profits, housing).

### 2.1. Literature search and selection criteria

The African continent covers 20.3% of the land area of then Earth this is 6% of the total surface of the planet. The continent has an area of 30,415,873 km^2^ (12), and Africans represent 16% of the world population.

For this study, we conducted systematic and advanced searches without language restriction using keywords on cancer costs in children in Africa in the following databases: Index Medicus African Health of the World Health Organization (AFROLYB, AIM, Global Health Library), PubMed, Cochrane Library, CISMeF, and Google Scholar. Additionally, we performed a standard search using search bots. We searched for article titles, summaries, reports, briefs, and any other electronic presentation for data on Africa without restriction on format type or year. The searched keywords were as follows: “cost of cancer”, “childhood cancer—socioeconomic factors”, “cancer financing in Africa”, “prospective study” and “African continent” using the logical separators AND (AND) and OR (OR).

The selected articles supported the evaluation of the cost of cancer in children aged 0–17 years in African countries. The inclusion criteria for the articles were (a) retrospective or prospective descriptive studies, (b) carried out in any type of health structure in sub-Saharan Africa between March 2000 and December 2022 in which the subjects were (c) children (d) with cancer regardless of type, younger than 17 years, (e) included descriptive information on pediatric cancer management; we did not include the different islands of the African continent in the study.

We also calculated the economic burden of childhood cancer at the individual level, by dividing the direct costs of cancer per patient by the GDP *per capita*, PPP of the country studied. This measure would indicate how catastrophic these costs could be for an average citizen (GDP *per capita*) (13).

GDP *per capita* based on purchasing power parity (PPP) is gross domestic product converted to international dollars using purchasing power parity rates. It is calculated without deduction for depreciation of manufactured assets or for depletion and degradation of natural resources. Data are expressed in constant 2017 international dollars (4). Cross-country comparisons based on market exchange rates of GDP to its expenditure components reflect both differences in economic output (volumes) and prices. Cross-country comparisons based on PPPs of GDP in its expenditure components only reflect differences in economic output (volume), because PPPs consider price level differences between countries. Therefore, the comparison reflects the actual size of the countries. The International Comparison Program (ICP) estimates PPPs for countries around the world (13).

### 2.2. Data extraction and analysis

We first selected articles based on their titles and then we searched the abstracts of those titles to screen focusing on one or more aspects of the financial cost of childhood cancer. Finally, we performed a manual search of the sources in the reference lists of articles we had selected that our online searches had not detected.

We also collected information such study: reference study, publication year, number of cancers, and direct and indirect cost data in US dollars.

The following information was collected for each study: study baseline, year of publication, number of cancers, and cost data in US dollars. The costs were estimated in US dollars, and findings were analyzed Stata 11.0 (StataCorp LLC). The direct cost of childhood cancer in Sub-Saharan Africa was calculated by geopolitical zone (Central, East, Southern, West Africa); and by type of cancer. The descriptive data are expressed in median and Interquartile range. The Kruskal Wallis rank sum test was performed for the direct cost of multiple groups. AP value <0.05 was the threshold.

To calculate the percentage burden of cost of pediatric cancer, the GDP *per capita* of countries where the studies were conducted was considered and converted into the international dollars by purchasing power party (2021).

### 2.3. Quality assessment

Methodological quality of retrieved articles was assessed using the 2022 CHEERS Checklist (14). The Consolidated Health Economic Evaluation Reporting Standards (CHEERS) statement, published in 2013, was created to ensure that health economic evaluations are identifiable, interpretable and useful for decision-making. The new 2022 CHEERS reporting replaces the previous CHEERS reporting guidelines. The checklist items are divided into seven main categories: (1) Title; (2) Summary; (3) Introduction; (4) methods; (5) Results; (6) Discussion; and (7) other relevant information (14).

## 3. Results

### 3.1. Selection of studies

**Figure 1:**
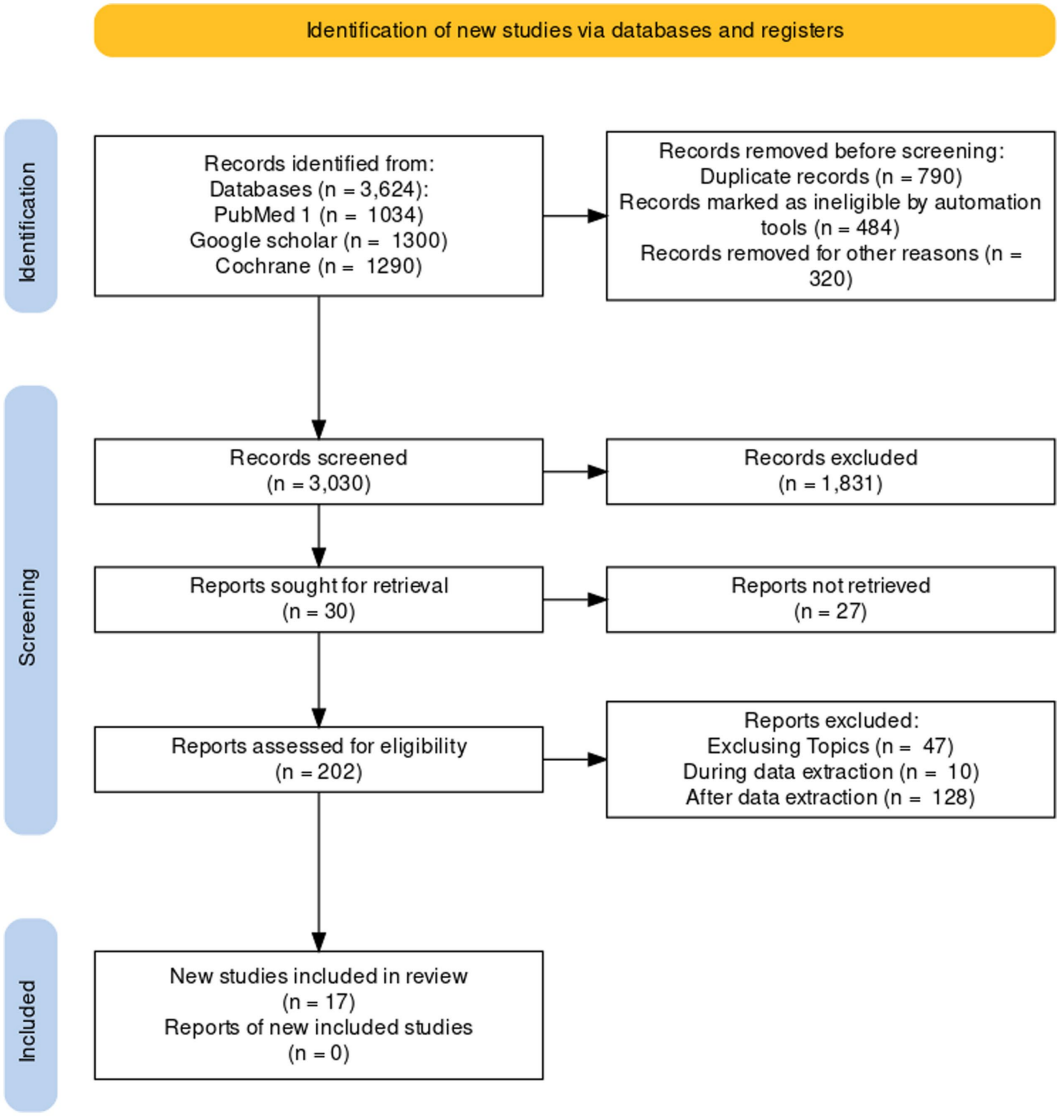
PRISMA diagram.

The number of abstracts of studies based on the Financial costs of pediatric cancer management identified from the databases was 3,624 (1,034 on PubMed, 1,300 on Google Scholar and 129 0 in the Cochrane Library). After adjustment 1,594 duplicates and 1,831 irrelevant were excluded. Of the 30 reports sought for retrieval, based on the review, three reports were retrieved and 27 were rejected. Of the 202 relevant articles assessed for eligibility, 184 did not meet the inclusion criteria (27 exlusing Topics, 5 during data extraction and 128 after data extraction). Seventeen articles fulfilling all the inclusion criteria were finally selected (PRIMA diagram Figure 1).

### 3.2. Study characteristics and methodological quality

The general characteristics of the studies on the financial costs of pediatric cancer management included are presented in Table 1. The first year of study was 2003 and the most recent year of study was 2021. Studies were primarily based on cost analysis (*n* = 12), CHEERS checklist score for each study. The median CHEERS checklist score was 19 out of 28 (16–22). The median (IQR) the direct cost associated with childhood cancer was 909.5 $ ($455.3–$1765).

**Table 1:** Study characteristics and methodological quality.

Areas	Author	Sample size	Type of economic analysis	Types of childhood cancer	Results (in $)			Cost included (US$)	Support protocols	CHEERS checklist (out of 28)
					Total cost	Direct cost (DC)	Indirect cost (IC)			
Sub-Saharan Africa										
Central Africa										
Democratic Republic of Congo (DRC)	Mjumbe et al.(15)	(129)^a^	THAT	Multiple childhood cancers				DC: $378.1 ± 61 CI: $146.3 ± 39 CT: 524.4 ± 50 Nephroblastoma: cost of $1,042 Leukemia ($977.3), Lymphoma ($831.7), Neuroblastoma ($803.1), Retinoblastoma ($797.5), Bone tumors ($733.8)	GFAOP	16
	Lukamba et al. (16)	(116)^a^	THAT	Retinoblastoma				Average cost per child: 1690 Lubumbashi: 2500 Kinshasa: 1490 Bukavu: 1080 Abudjan: 540	GFAOP	15
Cameroon	Israels et al. (17)	(255)^a^	THAT	Leukemias				CDs: 680.5	Adapted SIOP PODC treatment guideline	18
	Paintsil et al. (18)	(360)^a^	THAT	Wilms tumor				CDs: 416	Modified SIOP 2001	19
	Traoré et al. (19)	(178)^a^	THAT	Burkitt Lymphoma		799		Average cost of $685 per patient	GFAOP	14
West Africa										
Ghana	Dawson et al. (20)	(156)^b^	AC, AUC	Lymphoma	440.32	427.11	113.21	Average cost/month: 440.32, 97%	N/A	19
	Renner et al. (21)	(170)^a^	CA, CEA, AUC	Multiple childhood cancers		10,540		Together, medications, imaging, radiation, and pathology services accounted for 7% (119,000) Cost per child: 700 The cost per disability-adjusted life-year averted was $1,034	Adapted SIOP PODC treatment guideline	26
	Israels et al. (17)	(255)^a^	THAT	Leukemias				CDs: 680.5	Adapted SIOP PODC treatment guideline	22
	Paintsil et al. (18)	(360)^a^	THAT	Wilms tumor		1,110		CD: Total 1,100 US$	SIOP 2001	23
Ivory Coast	Lukamba et al. (16)	(116)^a^	THAT	Retinoblastoma		540		Average cost per child: 1690 Abudjan: 540	GFAOP	15
Burkina Faso, Ivory Coast, Mali and Senegal	Traoré et al. (19)	(178)^a^	THAT	Burkitt’s lymphoma		799		average cost of $685 per patient	GFAOP	14
Nigeria	Meremikwu et al. (22)	(41)^a^	THAT	Burkitt’s lymphoma		103.8		DC: 163.8 Initial diagnostic laboratory test: 18.9 Laboratory tests followed: 9.6 Drug cost: 103.8 Other medical expenses: 31.5	SIOP	19
	Joseph et al. (23)	(46)^a^	THAT	Multiple childhood cancers		13,876		Mean CD from diagnosis to remission or death: 13876 Rhabdomyosarcoma: 18678 Leukemia: 14,450		16
East Africa										
Rwanda	Neal et al. (24)	(66)^a^	THAT	nephroblastoma		1,913		Total cost Metastatic nephroblastoma: 2093	SIOP	26
				Hodgkin lymphoma		1,638		Total cost: 1793		
	Kanyamuhunga et al. (25)	(25)^a^	THAT	Leukemias		1,831.2		1,831.2 for early disease 2418.7 advanced disease	SIOP	23
Uganda	Denburg et al. (26)	(122)^a^	CA, CEA, AUC	Burkitt’s lymphoma		1,401		Average total cost: 4195 Variant cost: 1086.57 Fixed cost: 2646.54		26
	Paintsil et al. (18)	(360)^a^	THAT	Wilms tumor				CDs: 211		23
	Waddell et al. (27)	(270)^a^	THAT	Retinoblastoma				CDs: 1079		16
Tanzania	Saxton et al. (28)	(161)^a^	AC, AUC	Multiple childhood cancers		5,064 (IQ 4,746 to 5,501)		Total cost: 846743 Median cost: 5,064 (IQ 4,746 to 5,501) Direct drug cost: 664	SIOP	21
Ethiopia	Paintsil et al. (18)	(360)^a^	THAT	Wilms tumor		388			SIOP	23
Kenya	Githang’A et al. (29)		AC, CEA	Multiple childhood cancers				$31,344	SIOP	20
Southern Africa										
Zimbabwe	Githang’A et al. (29)		AC, CEA	Multiple childhood cancers				$2,338	SIOP	20
Madagascar	Traoré et al. (19)	(178)^a^	THAT	Burkitt’s lymphoma				799	GFAOP	14
Malawi	Paintsil et al. (18)	(360)^a^	THAT	Wilms tumor				122	SIOP	23
	Hesseling et al. (30)	(44)^a^	THAT	Burkitt’s lymphoma				217		16
	Israels et al. (17)	(255)^a^	THAT	Leukemias				680.5	the adapted SIOP PODC treatment guideline	22
South Africa	Stefan and Stones (31)	(138)^a^	THAT	Hodgkin’s lymphoma		7,360		CD: 6647.27/2 year	SIOP	22

CA, cost analysis; CEA, cost-effectiveness analysis; CUA, cost-utility analysis; IC, indirect cost; DC, direct cost; Median (IQR), Median (Interquartile range); GFAOP, Franco-African Pediatric Oncology Group; SIOP, International Society of Pediatric Oncology; PODC, Pediatric Oncology in Developing Countries.

a Child subjects only.

b Child and adult subjects.

**Figure 2:**
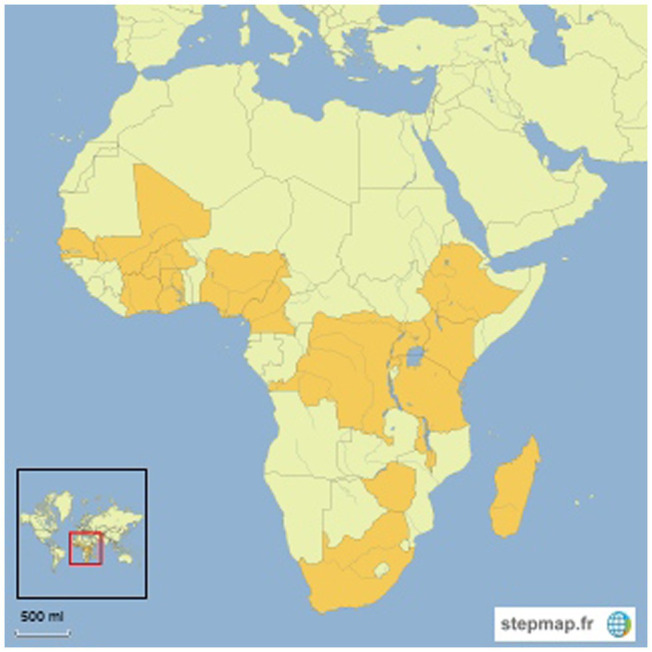
Countries represented in included studies.

However, nine studies were nationwide identified (22–28, 30, 31). Four studies were selected across regions (16–18, 29). Seven of 18 papers from East Africa five countries are represented the countries include Rwanda (24, 25). For Uganda we selected the following studies: Denburg et al. (26); Paintsil et al. (18); Waddell et al. (27). In Tanzania, we have following studies: Saxton et al. (28); Githang’A et al. (29). For Ethiopia, we have selected (18). In Kenya, we have selected (29). Six studies from Southern Africa, Zimbabwe (29), Madagascar (19), Malawi (17, 18, 30) and South Africa (31). Five of the articles came, respectively, from Central Africa, DR Congo (15, 16) and Cameroon (17–19); and West Africa, Cote d’Ivoire (16, 19), Ghana (17, 18, 20, 21), Burkina Faso, Mali, Senegal (19) and Nigeria (22, 23, 29) (Figure 2).

Only 2 out of 17 articles included (15, 20) reported both the average total cost, the direct cost and the indirect cost. Childhood cancer treatment costs were reported for lymphoma (*n* = 9), multiple cancers (*n* = 8), Wilms tumors (*n* = 5), leukemia (*n* = 4), retinoblastoma (*n* = 3), nephroblastoma (*n* = 1) and rhabdomyosarcoma (*n* = 1).

### 3.3. The direct cost of treating childhood cancer in sub-Saharan Africa

To explore the direct cost of childhood cancer in sub-Saharan Africa, we stratified costs by study region (Table 2). The direct cost of childhood cancer was lowest and highest in West Africa and Southern Africa, the two having $103.8–18,528$ [$799 ($540–$1960)] and $122–$7,360 [739.8$ ($332.9–$1542.3)] respectively. No significant difference (*p* < 0.05) was observed for comparisons of the direct cost related to childhood cancer between the Geopolitical zone sub-Saharan Africa (Central Africa, West Africa, East Africa and southern Africa).

**Table 2:** Pooled estimates of the direct cost of cancer treatment in Sub-Saharan Africa.

Geopolitical zone sub-Saharan Africa	*n* (%)	Median (interquartile range)	Min	Max	*p*-value
Central Africa	5 (17)	680.5 (416–799)	378.1	1,690	0.5956
West Africa	9 (30)	799 (540–1960)	103.8	18,528	
East Africa	10 (33)	1510.5 (1034.8–1782.9)	211	5,064	
Southern Africa	6 (20)	739.8 (332.9–1542.3)	122	7,360	

*p* < 0.05.

### 3.4. The direct cost of treating childhood cancer by type of cancer

All studies have differentiated costs by type of cancer, namely lymphoma (*n* = 9), multiple cancers (*n* = 8), Wilms tumors (*n* = 5), leukemia (*n* = 4) and other cancers [Retinoblastoma (*n* = 3), Rhabdomyosarcoma (*n* = 1), nephroblastoma (*n* = 1)] (Table 3). Differences in direct childhood cancer costs were significant for the different types of cancer (*p* < 0.05). Costs for leukemia patients ($1831.2) are significantly higher than costs for lymphoma patients ($103.8). The median cost (IQR) of patients with Wilms tumor was $388 ($211–$416). The associated median (IQR) costs of multiple cancers and other cancers (Retinoblastoma, Rhabdomyosarcoma, Nephroblastoma) were $1875 ($1,320–$7,802) and $1,690 ($1,079–$1913) respectively.

**Table 3:** Direct cost of cancer treatment by type of cancer.

Type of cancer	*n*	Median (interquartile range)	Min	Max	*p*-value
Multiple cancers	8	1875 (1,320–7,802)	378.1	13,876	0.0354
Wilms tumor	5	388 (211–416)	122	1,110	
Leukemias	4	680.5 (680.5–1255.9)	680.5	1831.2	
Lymphoma	9	799 (427.1–1,401)	103.8	7,360	
Other cancers	5	1,690 (1079–1913)	540	18,528	

*p* < 0.05.

The cost of cancer varied between $88,803.10 for neuroblastoma and $163.80 for lymphoma (Table 4). The Cost of Neuroblastoma Cancer, Bone tumors accounted for 68.09% (88803.10) and 62.21% (797.50) of DRC’s GDP respectively, in Nigeria 345.38% (18678) of Nigeria’s GDP for Rhabdomyosarcoma (22, 23). The cost burden was 67.61% (797.50) and 8.37% (500.00) for retinoblastomas in DRC and Ghana, each.

**Table 4:** Direct cost per childhood cancer according to gross domestic product *per capita* (GDP *per capita*), PPP.

Country	Study	GDP *per capita*, PPP (US $2021)	Components	Nephroblastoma	Leukemia	Lymphomas	Neuroblastoma	Retinoblastomas	Bone tumors	Wilms tumor	Multiple childhood cancers	Rhabdomyosarcoma
Democratic Republic of Congo (DRC)	Mjumbe et al. (15), Lukamba et al. (16)	1179.5	Cost per patient	1,042	977.3	831.70	88803.10	797.50	733.80	–	–	–
			% GDP *per capita*	88.34	82.86	70.51	68.09	67.61	62.21	–	–	–
Cameroon	Israels et al. (17); Paintsil et al. (18); Traoré et al. (19)	4065.3	Cost per patient	–	680.5	685.00	–	–	–	416.00	–	–
			% GDP *per capita*	–	16.74	16.85	–	–	–	10.23	–	
Ghana	Dawson et al. (20); Renner et al. (21); Israels et al. (17); Paintsil et al. (18)	5971.1	Cost per patient	–	680.5	5125.32	–	500.00	–	1100.00	700	–
			% GDP *per capita*	–	11.4	85.84	–	8.37	–	18.42	11.72	–
Ivory Coast	Lukamba et al. (16); Traoré et al. (19)	5850.1	Cost per patient	–		685.00	–	540.00	–	–	–	–
			% GDP *per capita*	–		11.71	–	9.23	–	–	–	–
Burkina Faso	Traoré et al. (19)	2394.7	Cost per patient	–		685.00	–	–	–	–	–	–
			% GDP *per capita*	–		28.60	–	–	–	–	–	–
Mali	Traoré et al. (19)	2329.7	Cost per patient	–		685.00	–	–	–	–	–	–
			% GDP *per capita*	–		29.40	–	–	–	–	–	–
Senegal	Traoré et al. (19)	3,840	Cost per patient	–		685.00	–	–	–	–	–	–
			% GDP *per capita*	–		17.84	–	–	–	–	–	–
Nigeria	Meremikwu et al. (22); Joseph et al. (23)	5,408	Cost per patient	–	14,450	163.80	–	–	–	–	13,876	18,678
			% GDP *per capita*	–		3.03	–	–	–	–	96.03	345.38
Rwanda	Neal et al. (24); Kanyamuhunga et al. (25)	2459.7	Cost per patient	2093	1831.2	1793.00	–	–	–	–	–	–
			% GDP *per capita*	85.09	74.45	72.90	–	–	–	–	–	–
Uganda	Denburg et al. (26); Paintsil et al. (18); Waddell et al. (27)	2467.9	Cost per patient	–	–	4195.00	–	1079.00	–	211.00	–	–
			% GDP *per capita*	–	–	169.98	–	43.72	–	8.55	–	–
Tanzania	Saxton et al. (28)	2836.2	Cost per patient	–	–	–	–	–	–		846,743	–
			% GDP *per capita*	–	–	–	–	–	–		29854.84	–
Ethiopia	Paintsil et al. (18)	2547.7	Cost per patient	–	–	–	–	–	–	388.00	–	–
			% GDP *per capita*	–	–	–	–	–	–	15.23	–	–
Kenya	Githang’A et al. (29)	5211.2	Cost per patient	–	–	–	–	–	–	–	31,344	–
			% GDP *per capita*	–	–	–	–	–	–	–	601.47	–
Zimbabwe	Githang’A et al. (29)	2232.7	Cost per patient	–	–	–	–	–	–	–	2,338	–
			% GDP *per capita*	–	–	–	–	–	–	–	104.72	–
Madagascar	Traoré et al. (19)	1607.9	Cost per patient	–	680.5	799.00	–	–	–	–	–	–
			% GDP *per capita*	–	42.32	49.69	–	–	–	–	–	–
Malawi	Paintsil et al. (30); Hesseling et al. (18); Israels et al. (17)	1638.2	Cost per patient	–	–	217.00	–	–	–	122.00	–	–
			% GDP *per capita*	–	–	13.25	–	–	–	7.45	–	–
South Africa	Stefan and Stones (31)	14624.4	Cost per patient	–	3323.635		–	–	–	–	–	–
			% GDP *per capita*	–	22.73		–	–	–	–	–	–

PPP, Purchasing Power Parity; GDP, Gross Domestic Product.

Regarding the cost of Nephroblastoma represents, respectively, 88.34% ($1,042) and 85.09% ($2,093) of the GDP of the DRC (15, 16) and Rwanda (24, 25). The cost of leukemia ranged between 11.4% ($680.5) in Ghana (17, 18, 20, 21) and 82.86% ($977.3) in DRC (15, 16). Lymphomas 3.03% (163.80) of Nigeria’s GDP (22, 23) and 85.84% ($5125.32) (17, 18, 20, 21) in Ghana.

## 4. Discussion

Our systematic review highlights the economic impact of childhood cancer as a burden in sub-Saharan Africa. Despite the increasing morbidity and mortality related to childhood cancer, data on its health care costs are limited in a resource-scarce health care environment like sub-Saharan Africa.

We performed a systematic review of studies reporting direct cost on Heinrich’s operational definitions (8–10). Studies aimed at improving outcomes for patients with cancers, particularly pediatric cancer should be measured not only in terms of epidemiological or clinical parameters, but also in terms of economic impact especially the impact felt by the household.

The World Health Organization (WHO) recommends that the results of COI studies be reported in international dollars at PPP, to better support country-to-country comparisons of costs (13). In the majority of 17 out of 54 countries on the continent based on the studies included in this review, the economic burden of childhood cancer is greater than 80% of GDP *per capita*, PPP (15–18, 20, 21, 24, 25), up to 345.38% of Nigeria’s GDP for Rhabdomyosarcoma (22, 23). Taking into account the definition of GDP *per capita* which corresponds to the average income of families (32), this is a cost that households cannot absorb without the support of the government or the various players. Pediatric cancer is therefore a real public health problem and an economic burden for households in at least 17 out of 54 countries on the continent based on the studies included in this review.

Assessing the economic costs of cancer to the health care system has its share of difficulties. Several researchers in the studies we identified reported high costs of cancer management in oncology units, the presence of which varied from country to country in Africa (33). In this area, the median direct cost (IQR) of childhood cancer was $909.5 ($455.3–$1765). Our analysis also showed that childhood cancer treatment costs ranged from $88,803.10 for neuroblastoma to $163.80 for lymphoma.

The direct costs of cancer treatment can be influenced by the complexity and availability of treatment (chemotherapy and/or surgery), the duration of chemotherapy, and the need for supportive care (34). Treatment complexity is generally lower for malignancies requiring only short-term chemotherapy regimens, such as Burkitt’s lymphoma and most lymphomas. Cancers requiring longer chemotherapy, such as acute lymphoblastic leukemia, or requiring surgery, such as Wilms tumor and retinoblastoma, are more complex, and cancers requiring surgery very complex (35).

In the Democratic Republic of Congo (DRC), for example, the highest cost was for retinoblastoma (1,690$) (16), although in three pilot treatment units (in the capital, Kinshasa; Haut-Katanga in Lubumbashi and Bukavu in the east), families can receive a loan of $1,419 per year (36). In neighboring Zambia and Rwanda, national subsidies for cancer patients significantly reduce the direct cost, to $49 and $61 per year (36).

Generally, the cost of care depends on the country, its standard of living and its health policy. Countries with an oncology-centric system pay 10 times the average of countries without government subsidies (7), and consistent with these findings, we found that cancer care in the Democratic Republic of Congo cost 10 times more than in neighboring countries, Rwanda and Zambia.

In Mauritania, a fixed price system was combined with user payment in public hospitals (37). The Rwandan oncology center observed a significant increase attendance when 90% of the cost of treatment was subtracted and a non-governmental organization funded treatment completely free (32).

Through these different methods of financing health systems, different countries have succeeded in guaranteeing real access to care in pediatric oncology (38). Results from a survey in Haiti found that use increased when care was free; free preventive care saw 2.87 times more patients than fixed price clinics with a price (39). In the DRC, however, there is no health insurance system or user fees, although support from the GFAOP is noted. Given this economic burden of childhood cancer, more research should be conducted on the costs of cancer care in Africa.

In our assessment of costing methods, we used the method CONGRATULATIONS 2022.

The main limitation of this systematic review concerns the quality of the existing literature in this area. Few of the economic evaluations in our study were of high methodological rigor, as evidenced by their scores on the CHEERS checklist. Future economic evaluations should adhere to the CHEERS Checklist, which consolidates previous economic evaluation guidelines and provides recommendations for optimizing the design and reporting of health economic evaluations (35).

The development of protocols for the economic evaluation of cancer should be thought out while taking into account the complexity and depend on the objectives of the studies. The protocols can contribute to reduce heterogeneity, by favoring the comparison between the different regions and the different health systems, in order to obtain a more precise calculation of the cost of oral cancer (…).

Several studies have underestimated the impact of non-medical indirect costs (8–10), in calculating the total cost of childhood cancer care. Only 2 (*n* = 2) out of 17 articles included (15, 20) have addressed both the notion of indirect and direct costs. We believe that the indirect cost results could be about the same in sub-Saharan Africa. Cancer represents a significant financial burden for families of children with cancer in Africa (15, 20). Families who pay to treat children with cancer are likely to suffer long-term economic and social repercussions related to debt repayment (32, 40–42).

Given the paucity of research on predictors of direct and indirect costs, researchers should explore other potential variables that may affect family costs, such as factors in the child’s illness, including physiological adaptation to his cancer and the side effects of the treatment; and social factors, including children’s absence from school. School absences are higher in children with cancer than in healthy children and those with other chronic diseases (43). Their absences are at all stages of their illness; however, they remain highest for the year following diagnosis. Thus, a lost school day due to frequent hospitalizations can also lead to a loss of parental productivity in the form of absenteeism or presenteeism, and can be an important predictor of costs (43).

Pediatric oncology units should include a well-established cancer registry and provisions to reduce the cost of care. Unfortunately, the situation in Africa is still far from ideal. Several countries in this region still do not have dedicated cancer units, and patients who are diagnosed with cancer face a sad fate, including a significant economic burden.

## 5. Conclusion

We identified with this systematic review we conclude that the economic burden of pediatric cancer care is very high in Africa, although we found significant heterogeneity in the 18 studies. When households have to pay for cancer care themselves, the cost is catastrophic, if not outright prohibitive. We believe that our findings are limited by the small number of countries that were represented and of studies on the costs of cancer care in Africa. We suggest that increasing knowledge on these topics would support making informed policies for financing health care systems in African countries.

Nevertheless, the data of our study which will be able to help to make different objective advocacy allowing to endow it with financial backer on the basis of the evidences which can reinforce this program in order to install in the country the structures of oncology and by following the plan of system cost reduction in the treatment of childhood cancer in particular and in general all types of cancer (adult). This program would be a valuable contribution to the existing employment insurance system and essential to ensure that households do not feel the great cost of cancer pathology; because, the fight is double financially and psychologically.

## Data availability statement

The original contributions presented in the study are included in the article/supplementary material, further inquiries can be directed to the corresponding author.

## Author contributions

CM and CD were responsible for the concept, design, and literature search of the study. MN, CM, and CD collected data. CM and CD performed the statistical analysis. MN, CM, CD, BI, DK, and ON drafted the manuscript. BI, DK, and ON supervised the study. CM, DK, ON, MN, DB, CD, and BI participated in the analysis and interpretation of the manuscript. All authors contributed to the article and approved the submitted version.
